# MRI-defined sacroiliitis and spinal inflammation in chronic back pain: real-world patterns in axial spondyloarthritis and axial psoriatic arthritis

**DOI:** 10.1093/rap/rkag072

**Published:** 2026-06-26

**Authors:** Mauricio Parada, María L Molina, Daniel Ríos, Daniela Suárez, Mauricio Ochoa, Natalie Hitchin, Cristóbal Bravo, Nicolás Guzmán, Pablo Barahona, Annelise Goecke

**Affiliations:** Rheumatology, Hospital Clínico de la Universidad de Chile, Santiago, Chile; Rheumatology, Hospital Clínico de la Universidad de Chile, Santiago, Chile; Radiology, Hospital Clínico de la Universidad de Chile, Santiago, Chile; Radiology, Hospital Clínico de la Universidad de Chile, Santiago, Chile; Internal Medicine, Hospital del Trabajador, Santiago, Chile; Medicine, Universidad de Chile, Santiago, Chile; Medicine, Universidad de Chile, Santiago, Chile; Medicine, Universidad de Chile, Santiago, Chile; Medicine, Universidad de Chile, Santiago, Chile; Rheumatology, Hospital Clínico de la Universidad de Chile, Santiago, Chile

**Keywords:** axial spondyloarthritis, psoriatic arthritis, chronic back pain, magnetic resonance imaging, sacroiliitis, inflammatory spinal lesions, diagnostic accuracy, real-world practice

## Abstract

**Objectives:**

To evaluate the diagnostic performance of MRI-defined sacroiliitis for axial spondyloarthritis (axSpA) in routine clinical practice and to characterize inflammatory spinal lesions (ISLs) and clinical factors associated with sacroiliitis across non-psoriatic axSpA (nPsA-axSpA), axial psoriatic arthritis (axPsA) and non-inflammatory chronic back pain (NI-CBP).

**Methods:**

We conducted a retrospective study of adults with chronic back pain who underwent SI joint and whole-spine MRI for suspected axSpA. Following longitudinal follow-up, patients were classified by expert rheumatologists as nPsA-axSpA, axPsA or NI-CBP. Two blinded musculoskeletal radiologists independently assessed MRI scans for sacroiliitis and ISLs using Assessment of SpondyloArthritis international Society/OMERACT definitions. Diagnostic accuracy metrics for MRI-defined sacroiliitis were calculated using expert diagnosis as the reference standard and multivariate logistic regression was used to identify predictors of sacroiliitis.

**Results:**

Ninety-five patients were included (29 nPsA-axSpA, 21 axPsA, 45 NI-CBP). MRI-defined sacroiliitis showed high specificity (91%) and moderate sensitivity (77–83%) for axSpA diagnosis. Independent predictors of sacroiliitis included age [odds ratio (OR) 1.05/year], regular physical activity (OR 3.50), expert-assessed inflammatory back pain (OR 35.11) and the number of SpA features (OR 1.72). ISLs were present in 40% of patients overall and were most prevalent in axPsA (76%). In axPsA, ISLs frequently occurred in the absence of sacroiliitis and displayed a broader and more heterogeneous distribution.

**Conclusion:**

MRI-defined sacroiliitis is highly specific but insufficient as a stand-alone diagnostic tool for axSpA in real-world settings and should be interpreted in context.

## Graphical Abstract

**Graphical Abstract:**
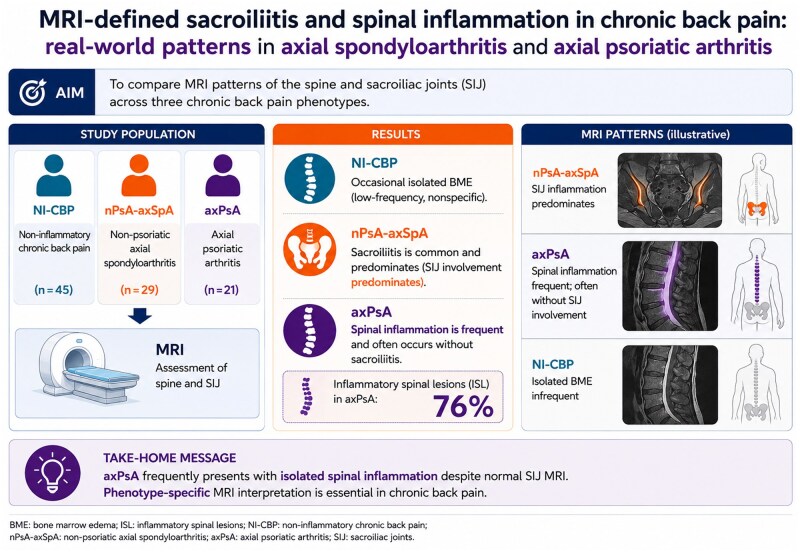

Key messages

- MRI-defined sacroiliitis is highly specific but insufficient alone to diagnose axial spondyloarthritis.
- Clinical probability and MRI findings must be integrated to avoid misclassification in chronic back pain.
- Axial psoriatic arthritis frequently shows spinal inflammation without sacroiliitis, requiring tailored MRI interpretation.

## Introduction

Axial spondyloarthritis (axSpA) comprises a heterogeneous group of inflammatory rheumatic diseases primarily affecting the axial skeleton, including the spine and sacroiliac joints (SIJs) [1–3]. Based on the presence or absence of structural damage on conventional pelvic radiographs, axSpA is classified into radiographic and non-radiographic forms [1]. Radiographic axSpA, corresponding to AS, is defined by structural SIJ changes that fulfil the modified New York criteria, whereas non-radiographic axSpA presents with similar clinical features but lacks radiographic evidence of structural damage [3].

axPsA is considered a distinct entity in which axial inflammation occurs in the context of psoriasis and may present with either radiographic or non-radiographic manifestations [4, 5]. All these conditions frequently present with chronic back pain (CBP) with or without clinical features of inflammation, posing a significant diagnostic challenge in differentiating them from non-inflammatory CBP (NI-CBP) [6]. Accurate distinction between inflammatory and mechanical back pain is crucial, given its implications for treatment decisions and long-term prognosis [7, 8]. To aid patient selection in axSpA research, the Assessment of Spondyloarthritis international Society (ASAS) proposed classification criteria in 2009 [1]. However, in routine clinical practice, the diagnosis remains based on comprehensive expert rheumatologic assessment [9, 10]. In this context, MRI plays a pivotal role in the early detection of inflammation in the SIJs and spine [11, 12]. According to the ASAS/OMERACT 2009 definition, a positive MRI for sacroiliitis is defined by the presence of bone marrow oedema (BME) visible on two or more consecutive slices or as two or more lesions on a single slice [11, 13]. MRI is particularly valuable for detecting early sacroiliitis, often before structural changes become apparent on radiographs. In the original validation of the ASAS criteria, MRI showed a sensitivity of 82.9% and a specificity of 84.4% for axSpA, with the imaging arm alone achieving a specificity close to 97% [1]. Nevertheless, interpretation of MRI findings without clinical correlation may lead to misclassification and thus unnecessary exposure to biologic therapies [14–16].

Despite being part of the axSpA spectrum, axPsA remains underrepresented in imaging studies, which have predominantly focused on AS [17]. While full-spine MRI adds little diagnostic value in AS [18], its utility in axPsA is less well established. NI-CBP represents a key differential diagnosis, given its high prevalence and the overlap of symptoms with inflammatory back pain (IBP) [19]. The presence of BME in patients with NI-CBP further complicates interpretation, highlighting the need for precise clinical–radiological integration [20].

In contrast to prior studies based on preselected cohorts fulfilling formal classification criteria, the present study was conducted in a real-world clinical setting, including patients with CBP who underwent SIJ and whole-spine MRI based on clinical suspicion of axSpA. Patients were subsequently classified by expert rheumatologists into non-psoriatic axSpA, axPsA or NI-CBP, reflecting everyday diagnostic scenarios in rheumatology practice [20, 21]

This approach allows evaluation of MRI findings across clinically relevant and diagnostically challenging subgroups rather than within trial-enriched populations. In particular, axPsA remains underrepresented in imaging studies, and the extent to which spinal inflammatory lesions contribute to its diagnosis, especially in the absence of sacroiliitis, remains unclear.

Therefore, the objectives of this study were to assess the diagnostic performance of MRI-defined sacroiliitis for axSpA using expert clinical diagnosis as the reference standard, identify clinical variables independently associated with sacroiliitis on MRI and describe the distribution and burden of inflammatory spinal lesions across non-psoriatic axSpA, axPsA and NI-CBP, with a specific focus on real-world diagnostic implications.

## Methods

### Study design and participants

A retrospective analysis was conducted using total spine and SIJ MRI scans performed at the Clinical Hospital of the University of Chile. This study included adult patients (≥18 years of age) presenting with back pain in whom MRI was indicated due to clinical suspicion of axSpA.

Patients were classified into three subgroups: non-psoriatic axSpA (nPsA-axSpA), axPsA and NI-CBP, according to the diagnosis established by an expert rheumatologist. Final diagnoses were based on comprehensive clinical assessment integrating clinical, laboratory and imaging data, including MRI findings, reflecting routine clinical practice.

To enhance the reliability of the reference standard, diagnostic classification was established through a structured review of each patient’s electronic medical record, requiring at least two follow-up rheumatology visits after the index MRI. This longitudinal evaluation allowed incorporation of disease evolution, additional clinical information and treatment response over time.

Only patients with sufficient post-MRI clinical data and at least two follow-up rheumatology visits were included. Cases lacking adequate clinical information or follow-up were excluded. A detailed flow diagram outlining the inclusion and exclusion process is provided in the supplementary material.

For the nPsA-axSpA and NI-CBP groups, only MRI scans performed in 2015 were included, whereas for the axPsA group, scans obtained between 2015 and 2021 were analysed. This extended inclusion period reflects the lower prevalence of axPsA in our clinical setting and was necessary to ensure an adequate and clinically representative sample.

### MRI protocol and image analysis

MRI scans were acquired using a 1.5T scanner without contrast. The acquisition protocol remained consistent throughout the study period. The protocol included fat-suppressed T1-weighted (T1w) and T2-weighted (T2w) sagittal sequences of the total spine, as well as T1w and short tau inversion recovery (STIR) semi-coronal sequences of the SIJs.

All MRI scans were independently evaluated by the same two experienced musculoskeletal radiologists, blinded to clinical data, and subsequently re-evaluated at a single time point to ensure consistent application of imaging criteria across all patients. In cases of disagreement, a consensus evaluation was performed to establish the final imaging classification used for analysis.

Sacroiliitis was defined according to the ASAS/OMERACT 2009 criteria, while inflammatory spinal lesions (ISLs), including spondylitis, spondylodiscitis, costovertebral joint arthritis, facet joint arthritis and enthesitis of spinal ligaments, were defined according to the ASAS/OMERACT 2012 criteria [11, 22]. ISLs were assessed at the patient level and each patient was categorized according to the number of distinct ISL subtypes present rather than per vertebral unit or spinal level. For the purpose of additional analyses, axial pain was categorized by anatomical region (lumbar, gluteal, thoracic and cervical) based on clinical records.

Topographic correspondence was defined descriptively as the presence of ASAS/OMERACT-defined inflammatory spinal lesions in the same anatomical region as the reported pain. Non-correspondence was defined as the presence of inflammatory lesions in a different spinal segment. As patients could present lesions in multiple spinal regions, corresponding and non-corresponding findings were not mutually exclusive. No formal agreement statistics were performed for this exploratory topographic analysis, given its descriptive nature.

### Clinical data and classification criteria

All patients were assessed using the 2009 ASAS classification criteria for axSpA [1]. For patients with axPsA, additional classification was performed using the Classification Criteria for Psoriatic Arthritis (CASPAR) [22]. Clinical features, including peripheral arthritis, enthesitis, uveitis, dactylitis, psoriasis, IBD, response to NSAIDs and family history of spondyloarthritis, were documented from electronic clinical records whenever available. Likewise, laboratory data, such as HLA-B27 status and CRP levels, were retrieved from electronic medical records when obtainable.

### Lifestyle factors

Physical activity was defined as engaging in at least 150 min/week of moderate–vigorous exercise, in accordance with the 2020 World Health Organization guidelines on physical activity and sedentary behaviour [23].

Female participants were classified as being in puerperium if they reported being pregnant within 6 months before the MRI scan. If this information was not available in their electronic medical record, patients were contacted by telephone to complete the missing data.

### Statistical analysis

Descriptive statistics were used to summarize patient and disease characteristics, with results reported as frequency (%) for categorical variables and mean (s.d.) or median [interquartile range (IQR)] for continuous variables, depending on data distribution.

For comparisons among subgroups, continuous variables were analysed using the Kruskal–Wallis test, followed by Dunn’s post hoc test with Bonferroni correction to identify pairwise differences, and categorical variables were compared using chi-squared (χ^2^) tests, and when expected cell counts were <5, Fisher’s exact test was applied. Post hoc pairwise comparisons for categorical variables were performed using χ^2^/Fisher’s exact tests with Bonferroni correction when global significance was detected.

Diagnostic performance metrics, including sensitivity, specificity, positive predictive value (PPV), negative predictive value (NPV) and likelihood ratios (LRs), were calculated using the epi.tests function from the R epiR package (version 4.4.1; https://cran.r-project.org/package=epiR).

Multivariate logistic regression analysis was performed in SPSS (version 21.0; IBM, Armonk, NY, USA) using the forward stepwise method (Wald test) to identify clinical variables independently associated with sacroiliitis. Potential collinearity among predictors was assessed and variables with high correlation were excluded to improve model stability. Model fit was evaluated using the Hosmer–Lemeshow test and discriminatory ability was assessed using the C-statistic (area under the curve).

Statistical significance was set at *P* < 0.05, with Bonferroni correction applied for multiple comparisons in post hoc analyses. All analyses were conducted in SPSS (version 21.0) and R (version 4.4.1).

Interobserver agreement for MRI-defined sacroiliitis and ISLs was assessed using Cohen’s kappa coefficient, with observed agreement also reported.

### Ethics approval

This study was approved by the Ethics Committee of the Clinical Hospital of the University of Chile (CECI-HCUCH; approval no. 051).

## Results

From an initial cohort of 257 SIJ and full-spine MRI scans, 162 were excluded due to incomplete clinical data at the time of MRI request (see Supplementary Fig. S1). The final sample comprised 45 patients with NI-CBP, 29 with nPsA-axSpA and 21 with axPsA. Clinical and demographic characteristics are summarized in Table 1.

**Table 1:** Demographic and clinical characteristics of the study participants.

Variable	All (*n* = 95)	NI-CBP (*n* = 45)	axSpA (*n* = 50)	nPsA-axSpA (*n* = 29)	axPsA (*n* = 21)	*P*-value
Age at MRI, years, mean (s.d.)	43.0 (13.3)	43 (15.3)	43 (11.3)	42 (11.3)	45 (11.2)	0.67
Female, *n* (%)	66 (69.4)	37 (82.2)	29 (58)	17 (58.6)	12 (57.1)	0.038^b^
Physical activity, *n* (%)	28 (29.5)	10 (22.2)	18 (36)	13 (44.8)	5 (23.8)	0.093
Smoking, *n* (%)	27 (28.4)	15 (33.3)	12 (24)	6 (20.7)	6 (28.6)	0.50
BMI, kg/m^2^, mean (s.d.)	25.5 (3.77)	25.2 (3.43)	25.7 (4.03)	24.2 (3.63)	27.8 (3.69)	0.0016^a^^,$^
Puerperium, *n* (%)	2 (3%)	1 (2.7%)	1 (2%)	1 (5.9%)	0	0.651
Mechanical back pain by expert, *n* (%)	68 (71.6)	42 (93.3)	26 (52)	16 (55.2)	10 (47.6)	<0.001^d^
IBP by expert, *n* (%)	27 (28.4)	3 (6.7)	24 (48)	13 (44.8)	11 (52.3)	<0.001^a^^,^^b^
IBP by ASAS, *n* (%)	11 (11.5)	2 (4.4)	9 (18)	5 (17.2)	4 (19.04)	0.117
Peripheral arthritis, *n* (%)	40 (42.1)	11 (24.4)	29 (58)	17 (58.6)	12 (57.1)	0.004^a^^,^^b^
Enthesitis, *n* (%)	40 (42.1)	11 (24.4)	29 (58)	17 (58.6)	12 (57.1)	0.004^a^^,^^b^
Anterior uveitis, *n* (%)	2 (2.1)	0	2 (4)	2 (6.9)	0	0.098
Dactylitis, *n* (%)	6 (6.3)	0	6 (12)	3 (10.3)	3 (14.3)	0.048
Psoriasis, *n* (%)	19 (20)	0	19 (38)	0	19 (90.5)	<0.001
IBD, *n* (%)	6 (6.3)	1 (2)	5 (10)	3 (10.3)	2 (9.5)	0.296
Family history of PsA or SpA, *n* (%)	6 (6.3)	0	6 (12)	2 (6.8)	4 (19)	0.012^a^
HLA-B27 positive, *n*/*N* (%)	5/26 (19.2)	1/8 (12.5)	4/18 (22.2)	3/10 (30)	1/8 (12.5)	0.3
CRP, mg/l, median (IQR)	8.0 (0.9–220)	7.4 (0.9–220)	8.5 (2.2–81)	8.0 (2.2–40.5)	12 (5–81)	<0.001^d^
axSpA by ASAS criteria, *n* (%)			28 (56)	18 (62)	10 (47)	0.13
Peripheral SpA by ASAS criteria, *n* (%)			28 (56)	18 (62)	10 (47)	0.13
SpA features^e^, median (IQR)	1 (0–2)	0 (0–1)	2 (1–3)	2 (1–2)	3 (2–3)	<0.001^d^
CASPAR, *n* (%)					13 (61.9)	
NSAIDs, *n* (%)	75 (78.9)	33 (73.3)	42 (84)	24 (82.8)	18 (85.7)	0.43
Corticosteroids, *n* (%)	20 (21.1)	3 (6.7)	17 (34)	15 (51.7)	2 (9.5)	<0.001^b^^,^^c^
csDMARDs, *n* (%)	40 (42.1)	0	40 (80)	21 (72.4)	19 (90.5)	<0.001^a^^,^^b^
bDMARDs, *n* (%)	20 (21.0)	1 (2.2)	19 (38)	11 (37.9)	8 (38.1)	<0.001^a^^,^^b^

csDMARD: conventional synthetic DMARD.

*P*-values indicate comparisons across all three subgroups.

HLA-B27 status was available for 26 patients.

a Significant difference between NI-CBP and axPsA.

b Significant difference between NI-CBP and nPsA-axSpA.

c Significant difference between axPsA and nPsA-axSpA.

d Significant difference among all three subgroups.

e SpA-related features correspond to the total number of classification items fulfilled per the ASAS criteria.

The mean age at the time of MRI scan was 43.0 years (s.d. 13.3, range 18–83) and 66 patients (69.4%) were female. The proportion of women was significantly higher in the NI-CBP group (82.2%) compared with those in the nPsA-axSpA (58.6%) and axPsA (57.1%) (*P* = 0.038) groups.

Physical activity was reported by 29.5% of patients overall, with subgroup proportions of 22.2% in NI-CBP, 44.8% in nPsA-axSpA and 23.8% in axPsA; however, this difference was not statistically significant (*P* = 0.093).

The mean body mass index (BMI) was 25.5 kg/m^2^ (s.d. 3.77), with significantly higher values in the axPsA group [27.8 kg/m^2^ (s.d. 3.69) compared with the other subgroups (*P* = 0.0016).

Puerperium was infrequent, observed in only 2 cases (3%), both within the NI-CBP and nPsA-axSpA groups.

Mechanical back pain was reported in 71.6% of the total cohort. It was significantly more frequent in the NI-CBP group (93.3%) but was also present in a substantial proportion of patients with nPsA-axSpA (55.2%) and axPsA (47.6%) (*P* < 0.001).

While 27 patients (28.4%) were classified by expert assessment as having IBP, only 11 patients (11.5%) met the ASAS IBP criteria (Table 1).

Smoking status did not differ significantly among groups (*P* = 0.50), although it was more prevalent in the NI-CBP group (33.3%) compared with the nPsA-axSpA (20.7%) and axPsA (28.6%) groups.

Interestingly, several patients in the NI-CBP group displayed features commonly associated with axSpA, such as enthesitis and peripheral arthritis (both in 24.4%). However, the total number of SpA-related features (per the ASAS classification criteria) was significantly lower in the NI-CBP group [median 0 (IQR 0–1)] compared with the nPsA-axSpA [median 2 (IQR 1–2)] and axPsA [median 3 (IQR 2–3)] (*P* < 0.001) groups.

Based on follow-up data from electronic medical records, 11 patients in the NI-CBP group were ultimately diagnosed with other rheumatic conditions: 3 with seronegative arthritis, 3 with seropositive RA, 3 with peripheral SpA and 2 with SS.

Anterior uveitis was reported exclusively in the nPsA-axSpA group (6.9%), while dactylitis, though infrequent, was only observed in the inflammatory groups: 10.3% in nPsA-axSpA and 14.3% in axPsA (*P* = 0.048).

Corticosteroid use (oral prednisone, intramuscular injection or joint infiltration within 3 months before or after the MRI) was significantly more common in the nPsA-axSpA group (51.7%) compared with the NI-CBP (6.7%) and axPsA (9.5%) groups (*P* < 0.001).

Regarding biologic therapy, only one patient in the NI-CBP group was receiving a biologic DMARD (bDMARD), but this patient had a confirmed diagnosis of seropositive RA, not an undiagnosed axSpA.

### Diagnostic performance of MRI-defined sacroiliitis for axSpA

To assess the robustness of imaging evaluation, interobserver agreement was analysed. Interreader agreement for MRI-defined sacroiliitis was moderate (Cohen’s κ = 0.56), with an observed agreement of 82.4%. For ISLs, agreement was fair (κ = 0.36), with an observed agreement of 74.8%.

MRI-defined sacroiliitis, based on ASAS/OMERACT 2009 criteria, was detected in 83% of patients with nPsA-axSpA, 76% of those with axPsA and only 9% of patients with NI-CBP. Using expert rheumatologist diagnosis as the reference standard, MRI demonstrated a sensitivity of 83% in the nPsA-axSpA group and 77% in the axPsA group, with a specificity of 91% observed in both cases.

Overall diagnostic accuracy measures, including PPV, NPV, positive LR and negative LR, for the combined axSpA population (nPsA-axSpA and axPsA) are summarized in Table 2.

**Table 2:** Diagnostic utility of MRI for sacroiliitis for the overall axSpA cohort.

Metric	Value (95% CI)
Sensitivity (%)	80.0 (66.0–90.0)
Specificity (%)	91.0 (79.0–98.0)
Positive likelihood ratio	9.0 (3.5–23.2)
Negative likelihood ratio	0.22 (0.13–0.38)
PPV (%)	91.0 (78.0–97.0)
NPV (%)	80.0 (67.0–90.0)

Diagnostic accuracy measures are presented with 95% confidence intervals (CI) using expert rheumatologist diagnosis as the reference standard for axSpA.

### Multivariate analysis of clinical variables associated with MRI-defined sacroiliitis

Multivariate logistic regression identified several clinical variables independently associated with MRI-defined sacroiliitis (Table 3). Age at the time of MRI was significantly associated with sacroiliitis, with each additional year increasing the odds by 5% [odds ratio (OR) 1.05 (95% CI 1.01, 1.11), *P* = 0.037]. Regular physical activity was also associated [OR 3.50 (95% CI 1.15, 10.62), *P* = 0.027]. Expert-assessed IBP showed a strong association with sacroiliitis [OR 35.11 (95% CI 2.01, 614.36), *P* = 0.015]. Moreover, the number of SpA features according to ASAS criteria was associated with increased odds of sacroiliitis [OR 1.72 per feature (95% CI 1.02, 2.90), *P* = 0.042]. BMI showed a non-significant trend [OR 1.15 (95% CI 0.99, 1.33), *P* = 0.059]. Other variables including sex, mechanical back pain and ASAS-defined IBP were not associated with sacroiliitis in the multivariate model.

**Table 3:** Multivariate logistic regression of clinical variables associated with MRI-defined sacroiliitis in the full study cohort (*n* = 95).

Covariates	Adjusted OR (95% CI)	*P*-value
Age at MRI (per year increase)	1.05 (1.01, 1.11)	0.037*
Sex (male *vs* female)	0.61 (0.19, 1.92)	0.366
BMI (kg/m²)	1.15 (0.99, 1.33)	0.059
Physical activity	3.50 (1.15, 10.62)	0.027*
Mechanical back pain by expert	7.23 (0.65, 79.91)	0.11
IBP by expert	35.11 (2.01, 614.36)	0.015*
IBP by ASAS criteria	7.23 (0.65, 79.91)	0.066
SpA-related features	1.72 (1.02, 2.9)	0.042*

* *P-*values <0.05 were considered statistically significant. The model was adjusted for collinearity; only variables with stable estimates were retained.

### Clinical differences by subgroup based on MRI-defined sacroiliitis

As shown in Table 4, patients with sacroiliitis on MRI tended to be older across diagnostic subgroups, with a significant age difference observed in the NI-CBP group (*P* = 0.049). BMI was also higher among patients with sacroiliitis across all groups, although this difference was not statistically significant. Physical activity was more frequently reported among patients with sacroiliitis in the axSpA group, a pattern not observed in NI-CBP. Notably, mechanical back pain as assessed by expert evaluation was reported in all NI-CBP patients with sacroiliitis and in more than half of axSpA patients with sacroiliitis. Expert-assessed IBP was significantly more frequent among axSpA patients with sacroiliitis, whereas this association was not observed when using ASAS-defined IBP, likely due to the small number of patients meeting the ASAS criteria.

**Table 4:** Clinical characteristics by diagnostic subgroup according to the presence or absence of sacroiliitis on MRI.

Variable	NI-CBP (*n* = 45)		nPsA-axSpA (*n* = 29)		axPsA (*n* = 21)		*P*-value
Sacroiliitis by ASAS/OMERACT criteria, *n* (%)	SI+: 4 (8.9)	SI−: 41 (91.1)	SI+: 24 (82.8)	SI−: 5 (17.2)	SI+: 16 (76.2)	SI−: 5 (23.8)	<0.001*
Age at MRI, years, mean (s.d.)	62 (4.6)	40.6 (14.6)	42.5 (11.3)	37.6 (11.7)	45.8 (11.49)	41.8 (10.6)	0.049*
Physical activity, *n* (%)	0	10 (24.4)	12 (50)	1 (20)	5 (31.3)	0	0.093
BMI, kg/m^2^, median (IQR)	26.6 (24.9–28.3)	24.2 (23.1–26.6)	24.7 (22.3–28.1)	20.3 (19.4–22.8)	29.5 (26.4–31.1)	23.5 (22.7–25.3)	0.127
Puerperium, *n* (%)	0	1 (2.4)	1 (4.2)	0	0	0	–
Mechanical back pain by expert, *n* (%)	4 (100)	38 (92.7)	13 (54.2)	3 (60)	8 (50)	2 (40)	<0.001*
IBP by expert, *n* (%)	0	3 (7.3)	11 (45.8)	2 (40)	8 (50)	3 (60)	0.018*
IBP by ASAS, *n* (%)	0	2 (4.9)	5 (20.8)	0	1 (6.3)	3 (60)	–
SpA-related features, median (IQR)	0 (0–0.5)	0 (0–1)	1.5 (1–2)	2 (2–3)	2.5 (2–3)	3 (2–4.5)	0.513
NSAIDs, *n* (%)	3 (75)	30 (73.2)	22 (91.2)	2 (40)	14 (87.5)	4 (80)	<0.001*
Corticosteroids, *n* (%)	0	3 (7.3)	11 (45.8)	4 (80)	0	2 (40)	0.043*
csDMARDs, *n* (%)	0	0	17 (70.8)	4 (80)	14 (87.5)	5 (100)	1.0
bDMARDs, *n* (%)	0	1 (2.4)	9 (37.5)	2 (40)	5 (31.3)	3 (60)	0.25

SI+: sacroiliitis positive; SI−: sacroiliitis negative.

* *P-*values correspond to Kruskal–Wallis test (continuous variables) or χ^2^/Fisher’s exact test (categorical variables). Post hoc comparisons were performed using Dunn’s test (for continuous variables) or pairwise χ^2^/Fisher’s exact test with Bonferroni correction (for categorical variables).

The number of SpA-related features according to ASAS criteria did not differ significantly between axSpA patients with and without sacroiliitis. Regarding treatment, NSAID use was significantly more common among axSpA patients with sacroiliitis, while corticosteroid use was more frequently reported in the nPsA-axSpA group with sacroiliitis.

### ISLs

ISLs were identified in 40% of the total study population, with a significant difference among diagnostic subgroups (*P* < 0.001; Table 5). The highest prevalence was observed in the axPsA group (76%), followed by the nPsA-axSpA (48%) and NI-CBP (18%) groups. The distribution of ISL subtypes is detailed in Table 5.

**Table 5:** ISLs) by diagnostic subgroup.

Variable	All (*n* = 95)	NI-CBP (*n* = 45)	nPsA-axSpA (*n* = 29)	axPsA (*n* = 21)
ISLs	38 (40)	8 (18)	14 (48)	16 (76)
Anterior/posterior spondylitis	20 (52.6)	4 (50)	7 (50)	9 (56.2)
Spondylodiscitis	3 (7.9)	1 (12.5)	1 (7.1)	1 (6.2)
Arthritis of costovertebral joints	12 (31.6)	0	3 (21.4)	9 (56.3)
Facet joint arthritis	15 (39.5)	1 (12.5)	5 (35.7)	9 (56.3)
Enthesitis of spinal ligaments	15 (39.5)	2 (25)	7 (50)	6 (37.5)
1 ISL	22 (23.2)	8 (17.8)	8 (27.6)	6 (28.6)
≥2 ISLs	16 (16.8)	0	6 (20.7)	10 (47.6)
≥3 ISLs	8 (8.4)	0	2 (6.9)	6 (28.6)

Values presented as *n* (%).

ISLs defined as the presence of active inflammatory findings on spinal MRI according to ASAS/OMERACT 2012 definitions. ‘1 ISL’, ‘≥2 ISLs’ and ‘≥3 ISLs’ refer to the number of distinct ISL subtypes identified per patient (spondylitis, spondylodiscitis, costovertebral joint arthritis, facet joint arthritis and enthesitis of spinal ligaments) rather than counts per vertebral unit or spinal level. A statistically significant difference in the prevalence of ISLs was observed across diagnostic subgroups (*P* < 0.001).

Among patients without MRI evidence of sacroiliitis, ISLs were detected in eight patients with NI-CBP and four with axPsA, while none were observed in the nPsA-axSpA group.

Notably, patients with axPsA demonstrated a higher number of distinct ISL subtypes per individual, with two or more lesion types identified in 10 patients compared with 6 in nPsA-axSpA and none in NI-CBP. Furthermore, three or more lesion types were observed almost exclusively in axPsA (6 of 8 cases).

To further explore the relationship between axial symptoms and imaging findings, an additional analysis of the anatomical distribution of axial pain and its spatial relationship with inflammatory spinal lesions was performed (Supplementary Table S1).

The topographic correspondence between axial pain and MRI-defined inflammatory lesions varied according to anatomical region. Lumbar pain demonstrated the highest degree of correspondence, particularly in the nPsA-axSpA subgroup, whereas gluteal pain showed a similar but less pronounced pattern. In contrast, thoracic and cervical symptoms exhibited weaker and more heterogeneous relationships with imaging findings across all groups.

In the axPsA subgroup, inflammatory spinal lesions were more frequently distributed across multiple spinal segments and showed a greater extent of non-correspondence with the symptomatic region compared with non-psoriatic axSpA. The distribution of structural lesions according to symptom location is presented in Supplementary Table S2.

## Discussion

In this real-world cohort of patients with CBP referred for MRI due to suspected axSpA, MRI-defined sacroiliitis demonstrated high specificity but only moderate sensitivity when expert clinical diagnosis was used as the reference standard. These findings confirm that, while SIJ MRI is a valuable diagnostic tool, its interpretation in isolation is insufficient and may lead to both under- and overdiagnosis when clinical probability is not adequately considered. This observation is consistent with prior studies reporting comparable diagnostic performance of MRI in routine clinical settings [24–27].

A key limitation of MRI-based sacroiliitis lies in the lack of specificity of BME, the hallmark lesion used in ASAS/OMERACT definitions. BME has been described in individuals with mechanical back pain, particularly in older patients and those with a higher BMI, even in the absence of inflammatory disease [28–31]. Accordingly, MRI findings must always be interpreted within the broader clinical context. In our cohort, age, BMI and physical activity showed trends toward an association with MRI-defined sacroiliitis, suggesting that common, non-inflammatory factors may act as confounders and increase the risk of false-positive interpretations [25, 32].

These findings align with previous reports indicating that up to 20–25% of healthy individuals or patients without clinical SpA may fulfil ASAS/OMERACT MRI criteria for sacroiliitis [33, 34]. Degenerative or age-related subchondral changes may further mimic inflammatory lesions, underscoring the diagnostic challenge of relying on imaging alone in patients without a clearly inflammatory phenotype [35].

The complexity of real-world diagnosis was further illustrated by the longitudinal follow-up of patients initially classified as NI-CBP. During follow-up, several patients were ultimately diagnosed with other inflammatory or autoimmune diseases, including seronegative arthritis, peripheral SpA, RA and SS. These patients frequently exhibited overlapping features such as peripheral arthritis or enthesitis that initially raised suspicion for axSpA. Importantly, only one of these patients demonstrated sacroiliitis on MRI, in the context of active RA. This highlights the difficulty of differentiating axSpA from other inflammatory conditions in routine practice, particularly when imaging findings are borderline [28, 36].

In our cohort, adherence to ASAS-defined IBP criteria was low, reflecting their limited sensitivity in heterogeneous clinical populations. Nevertheless, all patients ultimately diagnosed with axSpA presented with at least two to three characteristic SpA features. Given an estimated pretest probability of ≈5% for axSpA among patients with CBP, the combination of MRI-defined sacroiliitis (likelihood ratio ≈9) and multiple clinical SpA features may substantially increase post-test probability, supporting an integrated diagnostic approach that minimizes overdiagnosis and inappropriate treatment escalation [29–34].

Beyond sacroiliitis, our study highlights important differences in axial involvement across diagnostic subgroups. ISLs were observed in all groups but followed distinct patterns. Notably in our cohort, ISLs were not observed in patients with non-psoriatic axSpA in the absence of sacroiliitis, whereas a substantial proportion of patients with axPsA demonstrated spinal inflammation despite the absence of sacroiliitis. This finding supports emerging evidence suggesting that axial involvement in PsA may follow a different anatomical distribution compared with non-psoriatic axSpA [37–39].

To further explore the relationship between imaging findings and clinical presentation, we analysed the anatomical distribution of axial pain and its spatial correspondence with ISLs. We observed that the degree of topographic correspondence varied across spinal regions, with the strongest correspondence at the lumbar level, particularly in non-psoriatic axSpA. In contrast, thoracic and cervical symptoms showed weaker and more heterogeneous associations with MRI findings across all groups.

Notably, in axPsA, ISLs were more frequently distributed across multiple spinal segments and showed a higher degree of non-correspondence with the symptomatic region. This pattern may suggest a more diffuse and less anatomically consistent distribution of inflammation compared with non-psoriatic axSpA. These findings reinforce the concept that axial involvement in psoriatic disease may not follow the same topographic patterns as classical axSpA and highlight the limitations of relying on symptom location alone to infer the anatomical distribution of inflammation.

The burden and diversity of ISLs were greatest in axPsA, with multiple lesion types such as spondylitis, costovertebral joint arthritis, facet joint arthritis and ligamentous enthesitis frequently coexisting in the same patient. The presence of two or more lesion types, and particularly three or more, was observed almost exclusively in axPsA. Although limited by sample size, these findings are consistent with previous reports advocating for distinct imaging criteria in axPsA and align with hypotheses suggesting preferential involvement of ligamentous and posterior spinal elements in this phenotype [39, 40–42].

Consistent with prior literature, patients with axPsA in our cohort tended to be older, more frequently female and less often HLA-B27 positive than those with non-psoriatic axSpA. These demographic and imaging differences further support the concept of axPsA as a distinct clinical phenotype within the broader axSpA spectrum [4, 38, 39, 43]

At present, the lack of fully standardized MRI criteria specific to axPsA continues to limit diagnostic consistency and comparability across studies [37, 42]. Recent data from the AXIS cohort have provided important insights into the spectrum and distribution of axial involvement in PsA [43], supporting the concept of a distinct and heterogeneous phenotype. These findings may contribute to the development of more refined classification and imaging approaches, ultimately guiding more tailored diagnostic and therapeutic strategies [32].

The strengths of this study include the evaluation of MRI findings in a real-world clinical cohort encompassing NI-CBP, non-psoriatic axSpA and axial PsA, as well as the application of standardized ASAS/OMERACT definitions and the assessment of relevant clinical confounders. Importantly, all MRI scans were reinterpreted at a single time point by the same experienced musculoskeletal radiologists using standardized criteria, which reduces variability in image interpretation over time and enhances internal consistency across diagnostic groups. Interobserver agreement was moderate for sacroiliitis and fair for ISLs, consistent with the greater complexity and heterogeneity of spinal inflammatory findings in real-world imaging settings.

Several limitations should be acknowledged. The relatively small sample size, particularly in the axPsA subgroup, may limit statistical power and generalizability. The retrospective design and the requirement for complete post-MRI clinical data and follow-up may have introduced selection bias, potentially favouring patients with more structured clinical follow-up and diagnostic certainty. The asymmetrical inclusion periods between groups may also have introduced temporal heterogeneity and potential spectrum bias. Although MRI acquisition protocols remained stable, re-evaluation at a single time point minimized variability in interpretation.

In addition, because MRI forms part of the diagnostic work-up in suspected axSpA, the reference standard was not fully independent of imaging findings, introducing the possibility of incorporation bias and potential overestimation of diagnostic performance. Finally, the cross-sectional design does not capture longitudinal changes in inflammatory lesions.

In conclusion, while MRI-defined sacroiliitis remains a valuable diagnostic tool, it should not be used in isolation in patients with CBP. The frequent presence of inflammatory spinal lesions in the absence of sacroiliitis in axPsA underscores the need for phenotype-specific MRI interpretation and may support the development of tailored imaging criteria to improve diagnostic accuracy in routine rheumatology practice.

## Supplementary Material

rkag072_Supplementary_Data

## Data Availability

The datasets used and/or analysed during the current study are available from the corresponding author upon reasonable request.
