# Supplementary material for: MRI-defined sacroiliitis and spinal inflammation in chronic back pain: real-world patterns in axial spondyloarthritis and axial psoriatic arthritis
Source: Rheumatol Adv Pract. 2026 Jun 26;10(3):rkag072. doi: 10.1093/rap/rkag072 (PMC13456964; doi:10.1093/rap/rkag072)
Supplement: Supplementary file 1 [file rkag072_supplementary_data.docx]

**Supplementary Figure S1. Flow diagram of patient selection and cohort derivation**

**Supplementary Table S1**. **Topographic correspondence between axial pain and inflammatory spinal lesions across diagnostic subgroups**

| **Segment** | **Group** | **Pain n/N (%)^¶^** | **Corresponding ISL n (%)**^§^ | **Non-corresponding ISL n (%)^†^** | **No ISL n (%)** |
| --- | --- | --- | --- | --- | --- |
| **Lumbar** | NI-CBP | 30/45 (66.7) | 7 (23.3) | 3 (10.0) | 23 (76.7) |
|  | nPsA-axSpA | 18/29 (62.1) | 15 (83.3) | 4 (22.2) | 2 (11.1) |
|  | axPsA | 17/21 (80.9) | 14 (82.4) | 11 (64.7) | 1 (5.9) |
| **Gluteal** | NI-CBP | 16/45 (35.6) | 2 (12.5) | 1 (6.3) | 14 (87.5) |
|  | nPsA-axSpA | 12/29 (41.4) | 10 (83.3) | 2 (16.7) | 1 (8.3) |
|  | axPsA | 8/21 (38.1) | 8 (100.0) | 6 (75.0) | 0 (0.0) |
| **Thoracic** | NI-CBP | 9/45 (20.0) | 2 (22.2) | 0 (0.0) | 7 (77.8) |
|  | nPsA-axSpA | 12/29 (41.4) | 7 (58.3) | 10 (83.3) | 2 (16.7) |
|  | axPsA | 4/21 (19.0) | 2 (50.0) | 3 (75.0) | 1 (25.0) |
| **Cervical** | NI-CBP | 9/45 (20.0) | 1 (11.1) | 1 (11.1) | 8 (88.9) |
|  | nPsA-axSpA | 7/29 (24.1) | 4 (57.1) | 6 (85.7) | 1 (14.3) |
|  | axPsA | 2/21 (9.5) | 2 (100.0) | 2 (100.0) | 0 (0.0) |

§ Topographic correspondence was defined as the presence of inflammatory spinal lesions (ISL) in the same anatomical region as the reported pain.
† Non-correspondence was defined as the presence of inflammatory spinal lesions in a different spinal segment.

Inflammatory spinal lesions were defined according to the ASAS/OMERACT 2012 MRI consensus definitions and included spondylitis, spondylodiscitis, costovertebral joint arthritis, facet joint arthritis, and enthesitis of spinal ligaments.

¶ Percentages for pain are calculated based on the total number of patients in each subgroup. Percentages for corresponding and non-corresponding ISL are calculated based on the number of patients reporting pain in each anatomical region.

Corresponding and non-corresponding ISL are not mutually exclusive, as individual patients may present inflammatory lesions in multiple spinal segments. Therefore, percentages do not sum to 100%.

**Supplementary Table S2. Topographic distribution of structural lesions according to axial pain location across diagnostic subgroups**

| **Segment** | **Group** | **Pain n/N (%)^¶^** | **Corresponding**  **Structural lesions, n (%)**^§^ |
| --- | --- | --- | --- |
| **Lumbar** | NI-CBP | 30/45 (66.7) | 11 (36.7) |
|  | nPsA-axSpA | 18/29 (62.1) | 8 (44.4) |
|  | axPsA | 17/21 (80.9) | 2 (11.8) |
| **Gluteal** | NI-CBP | 16/45 (35.6) | 6 (37.5) |
|  | nPsA-axSpA | 12/29 (41.4) | 3 (25.0) |
|  | axPsA | 8/21 (38.1) | 0 (0.0) |
| **Thoracic** | NI-CBP | 9/45 (20.0) | 2 (22.2) |
|  | nPsA-axSpA | 12/29 (41.4) | 5 (41.7) |
|  | axPsA | 4/21 (19.0) | 1 (25.0) |
| **Cervical** | NI-CBP | 9/45 (20.0) | 3 (33.3) |
|  | nPsA-axSpA | 7/29 (24.1) | 4 (57.1) |
|  | axPsA | 2/21 (9.5) | 0 (0.0) |

§ Structural lesions refer to chronic MRI changes (e.g., sclerosis, fat metaplasia, or structural damage) and were analyzed separately due to their distinct pathophysiological significance compared with inflammatory lesions. Structural correspondence was defined as the presence of structural lesions in the same anatomical region as the reported pain.

¶ Percentages for pain are calculated based on the total number of patients in each subgroup. Percentages for corresponding structural lesions are calculated based on the number of patients reporting pain in each anatomical region.
